# Homemade Diet as a Paramount for Dogs’ Health: A Descriptive Analysis

**DOI:** 10.3390/vetsci11090438

**Published:** 2024-09-17

**Authors:** Giulia Pignataro, Paolo Emidio Crisi, Elena Landolfi, Benedetta Belà, Isa Fusaro, Luana Clerico, Alessandro Gramenzi

**Affiliations:** 1 Department of Veterinary Medicine, University of Teramo, 64100 Teramo, Italy; pecrisi@unite.it (P.E.C.); platobio31@gmail.com (B.B.); ifusaro@unite.it (I.F.); agramenzi@unite.it (A.G.); 2 Independent Researcher, 17100 Savona, Italy

**Keywords:** homemade diet, dog nutrition, follow-up study, gastroenteropathy, dermopathy

## Abstract

**Simple Summary:**

It is a fact that a pet’s health is significantly influenced by proper nutrition. Pet owners increasingly seek support for cooking human-grade fresh food for their animals, driven by their concerns about commercial food quality and the rising prevalence of diet-related diseases. This shift in pet care practices provides nutritionists with an excellent opportunity to prevent future diseases or enhance the treatment of sick pets through customized homemade diets. Despite promising clinical data, the bibliography must make more scientific evidence or guidelines available. The present study contributes to this goal by presenting favorable results on a group of dogs that experienced improved wellbeing and reduced symptoms following a homemade dietary plan. Particularly impressive outcomes have been observed in dogs with gastrointestinal and dermatological pathologies. These findings underscore the potential benefits of personalized and balanced homemade diets, paving the way for further studies to explore this area.

**Abstract:**

Pet nutrition awareness has risen enormously, with an increasing interest in homemade diets aimed at controlling food composition. The literature in this field is scarce, especially regarding the homemade diet’s long-term effects on pathological conditions. This prospective study encompassed 167 healthy and sick dogs subjected to a customized dietary plan based on homemade food. After an initial visit, dog owners provided questionnaires with follow-up data on their perceptions of physical sign changes or symptom modifications. A total of 104 (62%) subjects maintained the homemade diet, while 63 reverted to their previous diet. The median follow-up was 14 months. Out of 31 healthy dogs that continued the nutritional plan, 70% exhibited improvements in their coat condition and 47% a decrease in evacuation frequency. Regarding weight loss goals, 67% of dogs achieved their target. The 67 pathological dogs that completed the follow-up were primarily affected by gastrointestinal and dermatological disorders. Dogs with chronic enteropathy improved their symptoms in 95% of the cases, subjects with dermatological pathologies in 83%, and patients presenting both disorders in 100%. These clinical results are promising. Personalized and well-balanced homemade diets noticeably enhanced the overall pet’s health, with an almost complete remission of symptoms in pathological dogs.

## 1. Introduction

Over the past few decades, there has been a remarkable increase in pet owners’ consciousness regarding the health, wellbeing, and nutritional requirements of their animals [1]. This heightened awareness has prompted considerable attention to ensure that pets receive optimal nutrition, as it is pivotal in elevating their overall quality of life [2,3,4]. 

The desire for greater control over food composition and belief in providing a healthier and more natural approach are among the key factors contributing to the preference for homemade diets. Homemade diets can be tailored to meet the specific nutritional needs of individual dogs, potentially leading to improved health outcomes. Additionally, preparing homemade diets fosters a profound emotional bond between pet owners and their animals, even if it requires time, effort, and knowledge of canine nutrition [2,3,4].

Moreover, the mounting concerns about the quality and safety of ingredients used in commercial pet foods [3] have been further amplified by non-scientific online sources linking such diets to chronic pet illnesses. This surge in worry has led many pet owners to seek alternative feeding options to ensure the wellbeing of their pets. 

This trend underscores the crucial role of veterinary practitioners, who are now experiencing increasing inquiries about alternative diets and requests for guidance on homemade feeding [5]. It is now of utmost importance for veterinarians to be well-informed and knowledgeable on this subject, enabling them to offer accurate and comprehensive explanations to pet owners, direct them to nutritionists, and thereby discourage reliance on unverified sources lacking scientific validity.

Utilizing home diets extends beyond proactive health-conscious choices; it has also become a viable approach within clinical practice, demonstrating its potential benefits. For instance, veterinarians may opt for a homemade diet to assess the patient’s response to dietary changes when diagnosing food allergies or managing chronic intestinal diseases [6]. Moreover, in cases involving patients with decreased appetite or those suffering from multiple diseases, home diets can prove instrumental in stimulating appetite and providing tailored nutritional solutions [7]. 

The primary objective of homemade diets revolves around promoting dietary diversity, which in turn facilitates the cultivation of a healthy and varied gut microbial flora [8]. In the long run, this approach may mitigate the risk of developing pet food intolerances or allergies [9].

Nevertheless, few publications demonstrate the benefits of the homemade diet. In veterinary nutrition, the long-term effects of this diet on the animal’s quality of life and clinical success in pathological animals have yet to be investigated. This fact underscores the need for further research in this area, highlighting the crucial role of experts in animal nutrition in advancing the understanding of homemade diets and their potential benefits.

For these reasons, the main objective of this experimental study was to explore, over a period of time, dog owners’ perceptions of those who sought nutritional consultation for homemade diets for various purposes. Furthermore, the study aimed to investigate the prevalence of owners who consistently maintained this dietary approach versus those who abandoned it.

## 2. Materials and Methods

One hundred and sixty-seven dogs who underwent nutritional counseling from October 2020 to May 2022 were enrolled in this prospective study. Dogs were enrolled consecutively regardless of sex, age, and breed. Their owners had to choose a homemade maintenance diet to be included in this study. Both healthy and diseased dogs were included.

**Scheme 1:**
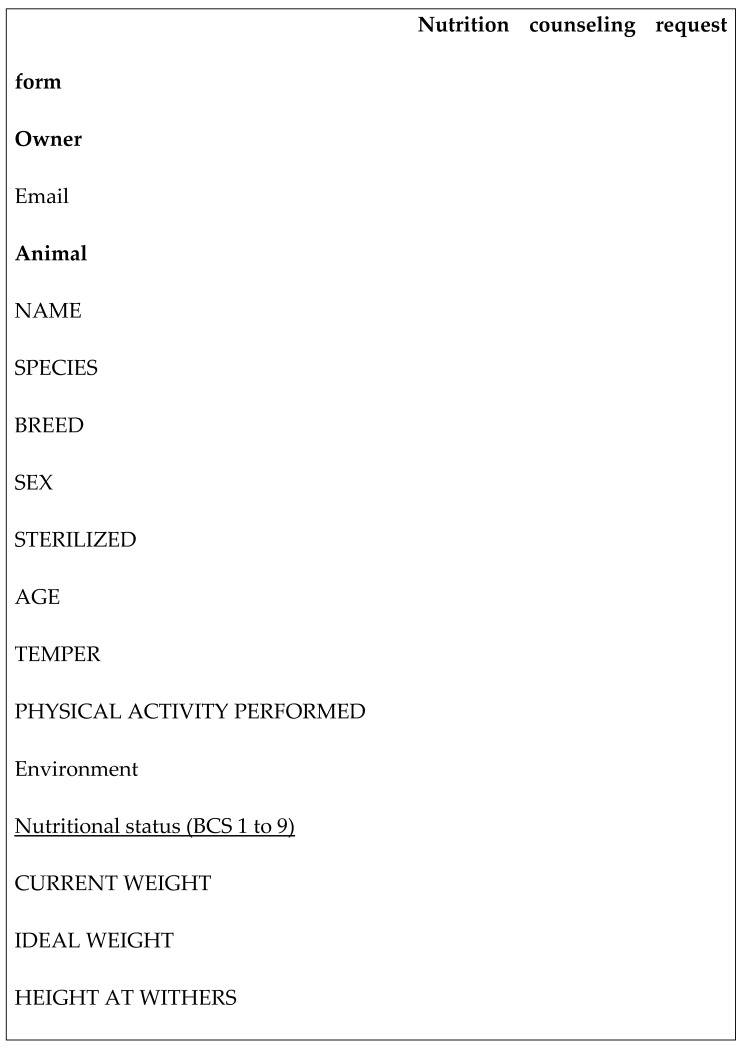

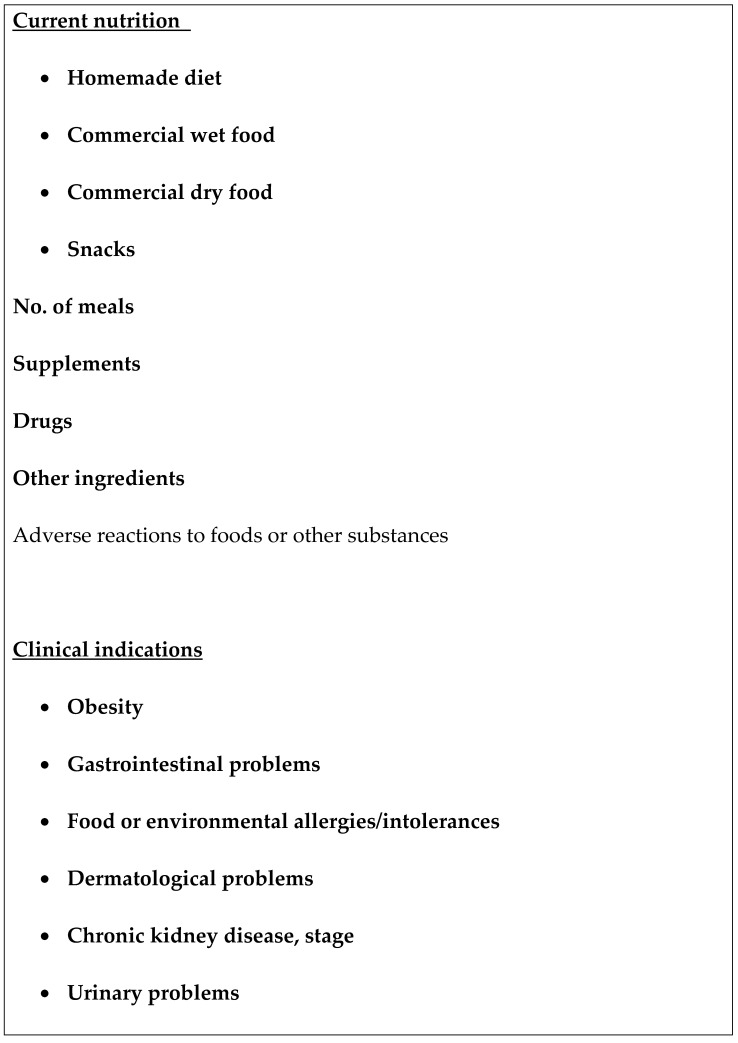

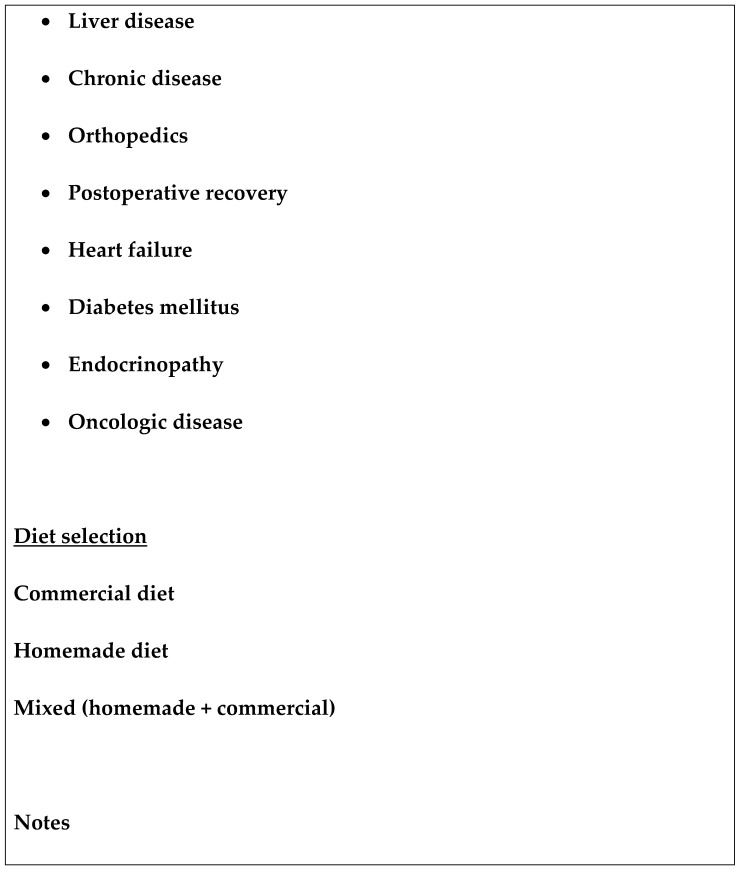
Copy of the record filled during the veterinary and nutritional visit.

During the initial veterinary visit, a comprehensive nutritional medical record was compiled (see Scheme 1), capturing all the information about the animal’s lifestyle and recent and remote medical history, with particular attention to food habits and allergies. This record also included additional details about ongoing or prior treatments in cases with underlying diseases. This comprehensive approach ensured that no aspect of the dog’s health and diet was overlooked.

Each dog was provided with a personalized nutritional plan (software PetDiet Pro vers. 2.7.2, veterinary diet balancing program) consisting of fresh human foods integrated with essential vitamin–mineral supplements (Carevit Ultra Pet^TM^, NBF Lanes, Milan, Italy) to ensure the proper balance of micronutrients in the diet. Each food plan was formulated according to the dog’s specific nutritional requirements, individual preferences reported by the owner, any existing allergies or intolerances, and the specific medical conditions necessitating the consultation.

Homemade diets primarily use specific protein sources such as chicken or turkey breast, pork loin, frozen cod fillets, or veal. Carbohydrate sources include rice, potatoes, and couscous. Vegetable fats are mainly sunflower seed oil, while animal fats are salmon oil. Vegetables such as zucchini, carrots, or legumes like peas are also included.

All the food used for the diets was human-grade and could be purchased at regular supermarkets. The recommended cooking methods were steaming or boiling in a small quantity of water, ensuring the preservation of nutrients. It was suggested that the meal be fed twice a day.

In cases of obesity, low-calorie diets were designed to achieve a target weight loss of approximately 1–2% per week. For subjects with pathological conditions, selecting functional foods was predicated on the specific pathologies observed and the ongoing therapies. This approach encompassed the incorporation of pathology-specific nutraceutical complementary feeds. In subjects with food allergies, new highly digestible protein and carbohydrate sources, hitherto unutilized by the animals, were considered.

At the end of the study period, the dogs’ owners were asked to complete a questionnaire to collect follow-up data. Owners received the questionnaire via email. Each digital questionnaire was specific for each patient, whether the diet had been formulated for a healthy or a pathological dog. The latter case focused mainly on dogs with dermatopathy and gastroenteropathy, and there were mixed cases of the two conditions.

To ensure the study’s robustness, the owners of the enrolled dogs were requested to provide detailed information on various aspects of their dog’s health and wellbeing. This comprehensive data collection encompassed improvements in ration palatability, coat and stool quality, defecation frequency, body conditions score (BCS), and activity level. They were also asked to report any adverse reactions or the occurrence of diseases. 

If the owners decided to discontinue the home diet, they were also asked to report the reasons for returning to the previous diet.

If the new dietary intervention was requested for disease-related conditions, further questions focused on the progress of the disease and whether the diet had improved the symptomatology were asked.

In addition, the questionnaires also left space to report whether the supplements and nutraceuticals in the diet were discontinued and had encountered problems in their administration.

The data collected were obtained anonymously, following the provisions of European Regulation 2016/679 (General Data Protection Regulation) concerning the protection of personal data, after the participants consented to processing their personal and sensitive data for this research.

All responses were considered valid only after the participants accepted the consent to process sensitive data and correctly completed the questionnaire.

A Fisher’s exact test was used for dichotomous data, while questionnaire responses were reported as percentages. 

We also used a logit regression to test the significance and impact of continuing a home diet versus ending it early. In particular, we decided to use this model, as it allows us to analyze categorical and dichotomous variables. The variables analyzed were binary and categorical: appetite (not improved; improved), coat (not improved; improved), energy (not improved; improved), stool frequency (not improved; improved), and consistency (not improved; improved). The independent variables included as control variables were dog size, age, sex, and the home-cooked diet dichotomous variable.

## 3. Results

In the clinical study, 167 dogs of both sexes, small, medium, and large in size, aged between 7 months and 14 years, were included. Fifty dogs (30%) were half-breed, and 117 (70%) belonged to purebred breeds. Of the 167 dogs, 74 females were neutered (75% of the females), while 39 males were neutered (57%).

**Figure 1:**
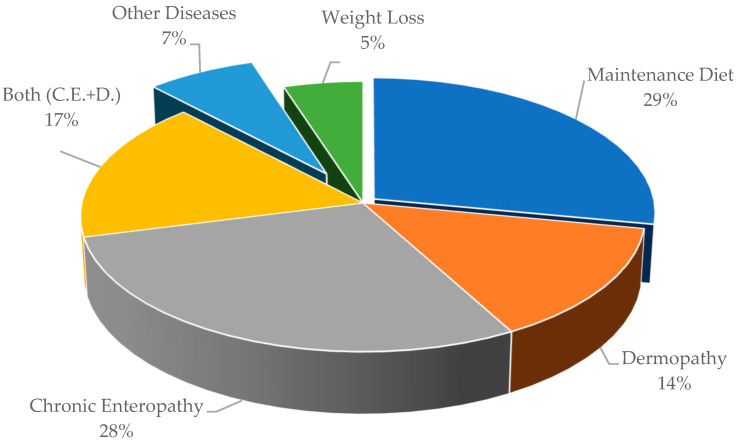
Case stories of referrals for nutritional consultation and formulation of homemade diet.

Referrals for nutritional consultation were divided into distinct categories: 48 cases were for a maintenance diet, 9 for obesity, 47 for chronic gastroenteropathy, 23 for dermopathy, and 29 for both gastrointestinal and dermatological conditions, with the remaining 11 cases also involving other pathologies (Figure 1).

The median follow-up, derived as the time between the nutritional counseling and the last contact, was 14 months (range 5–24).

At the time of the nutritional consultation, 67% of the dogs were on commercial pet food diets while only 17% were on exclusive balanced homemade diets, and the remainder (16%) were on mixed diets. 

The percentage increase in homemade diet use at the end of the study was statistically significant (*p* < 0.0001), with 104 out of 167 dogs (62%) maintaining the homemade diet until the end of the study. In contrast, 63 (38%) reverted to their previous diet (Table 1). The primary reasons for discontinuing the prescribed nutritional plan were predominantly linked to owner compliance (27 cases). Other causes of discontinuation were attributed to the dog’s failure to adapt to a new dietary regimen (15 cases), lack of palatability (8 cases), occurrence of new diseases (5 cases), and the death of the animal for reasons unrelated to nutritional issues (8 cases). 

It should be noted that among the 104 dogs who completed the follow-up while keeping the personalized and balanced nutritional plan formulated according to their unique needs and preferences, 80 (48%) ate only a homemade diet cooked with fresh foods, while 24 subjects (14%) were fed a mixed diet, i.e., a mix of homemade diet and a lower percentage (around 20–30%) of commercial food.

Table 1 describes the percentage increase in homemade diet use at the end of the study.

Table 2 enumerates the reasons for nutritional consultations in both the subjects who completed the follow-up and the ones who did not complete the study, showing that the two groups were largely comparable.

**Figure 2:**
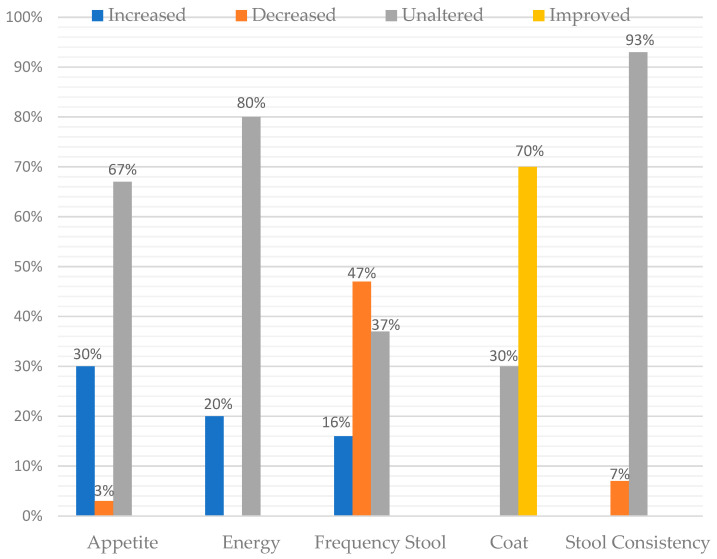
Results of the 31 subjects who used a homemade diet for maintenance purposes.

Among the 48 healthy patients for which the homemade diet was requested for maintenance purposes, 31 dogs continued the nutritional plan, exhibiting, according to owners’ perception in 70% of cases, improvements in their coat condition, which became shinier and softer. Moreover, 47% of them experienced a decrease in defecation frequency. Parameters such as stool consistency, appetite, and energy levels remained relatively stable in this subgroup of dogs (Figure 2). Nevertheless, in five subjects, symptoms emerged after introducing the new food plan, primarily manifesting as gastrointestinal disturbances.

**Figure 3:**
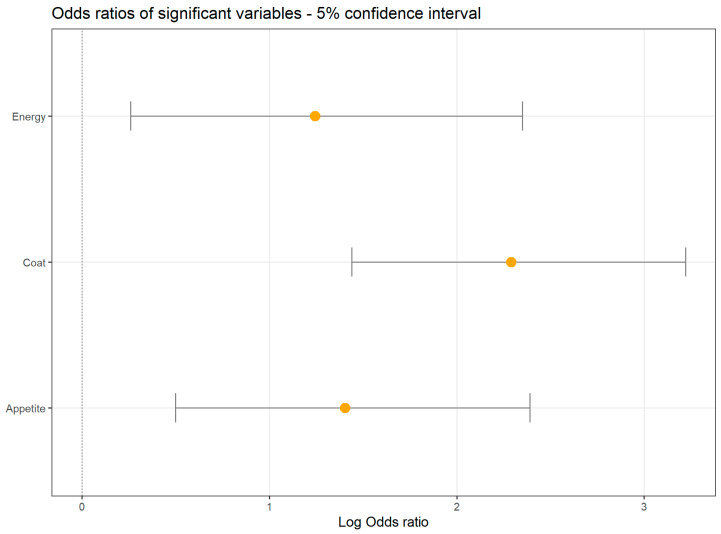
Graphic of the odds ratios of significant variables in terms of energy (logs 1.244), coat (logs 2.291), and appetite (logs 1.404).

We analyzed palatability, coat, energy, and frequency and consistency of the feces. Specifically, this latter variable was defined as follows: 0 was having finished the home-cooked diet early; 1 was having completed it. The odds ratios of significant variables are presented in the following Figure 3. We decided to present the odds ratios, as they allow us to better explain the impact of the estimated coefficient (Table 3).

Among the six dogs with weight loss goals that maintained the homemade diet, four achieved the set objective (67%), attaining an appropriate BCS. These four dogs were without overt symptoms, so the scope was preventive. They were small (one dog), medium (one dog), and large dogs (two dogs), young and old (range 2–12 years), all neutered, with different levels of activity. The common condition was due to the fact that all of them were previously fed mainly with commercial food. The remaining two dogs did not reach the weight target at the end of the study period. 

Among the 104 subjects that completed the follow-up, 67 (64%) were pathological dogs primarily affected by gastrointestinal and dermatological disorders. 

Specifically, the 43 subjects with chronic enteropathy had chronic diarrhea in 22 cases (51%), while vomiting was observed in 7 cases (16%) and reflux in 4 (9%). Apart from the presence of three puppies, the 27 studied dogs (75%) with this pathology were either young or adults. 

Regarding the 12 subjects with dermatological disorders, six cases were diagnosed with dermatitis (50%), while three (21%) experienced otitis and two (17%) presented with mono- or bilateral lacrimation.

**Figure 4:**
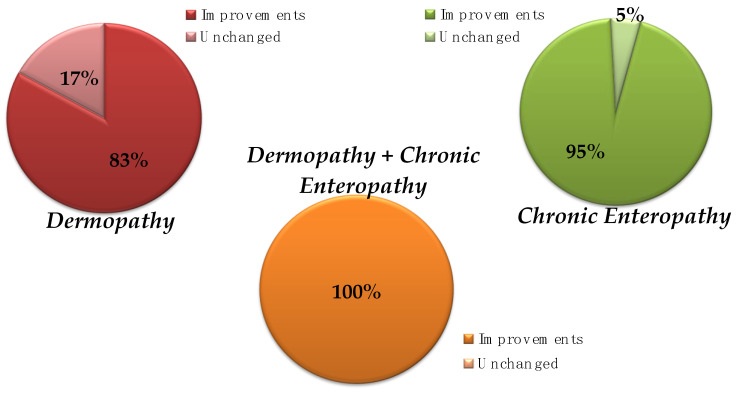
Homemade diet results obtained from 43 subjects with gastrointestinal conditions, 12 with dermatological conditions, and 12 with a combination of both conditions.

As shown in Figure 4, the owners reported that the dogs affected by chronic enteropathy mainly benefited from a personalized homemade diet, improving their symptoms in 95% of the cases. Dogs with dermatological pathologies reached significant improvements in 83% of the cases. Moreover, the patients presenting with both gastrointestinal and dermatological disorders at the beginning of the study dramatically improved their symptoms in all cases.

All formulated nutritional plans also included the administration of complete supplementation for vitamins and minerals, while specific supplements were additionally prescribed for pathological cases depending on the clinical presentation. The follow-up revealed that the supplementation was well tolerated in 79% of the cases. Nevertheless, 21% of dog owners ceased providing the supplements. The main reasons for the suspension were because supplements were unpalatable to the dog (93%) or challenging to find (7%).

## 4. Discussion

The motivations for approaching a homemade diet identified in this study, such as the desire for greater control over food composition and the belief in providing a healthier and more natural approach, have also been documented in the existing literature [2,3,10]. 

The role of veterinarians, pet nutritionists, and researchers is crucial in the success of a nutritional plan. Monitoring the animal over time not only helps assess the owner’s compliance but also evaluates the efficacy of the nutrition plan being drawn up or adjusted.

The starting point of this study concerning the rising request for homemade diets aligns with previous research and is consistent with the increasing awareness among pet owners regarding the importance of pet nutrition and wellbeing [2,3,4]. 

The finding that 67% of the dogs at the beginning of this research were on commercial pet food diets while only 17% were on exclusive homemade diets suggests that despite the increasing interest in homemade diets, commercial pet foods remain the dominant choice for most dog owners [11]. This preference could be mainly due to the increased costs associated with fresh ingredient and supplement purchases compared to commercial pet food. Additionally, the considerable effort and time in preparing the homemade ration could contribute to the choice [7].

Significantly, this study’s findings underscore the potential benefits of a tailored and balanced homemade diet [12] or a prevalence of homemade diet mixed with a lower percentage of commercial pet food. These findings reveal that such diets brought satisfaction to dog owners in 62% of the cases in the long run, with a satisfaction rate equal to or close to 100% in the owners of animals with dermatological and gastrointestinal underlying diseases. 

Statistically, it was found that in pure GI cases or in association with dermopathy, but not in pure dermatopathy, the dogs returned more easily to the old diet. This may of course be attributed to the finding that they are not food responders, but it also underscores the need to persist with the diet if it fails. 

However, when investigating satisfaction in dogs who completed follow-up, it was higher for the owners of the enteropaths than for the dermatopaths. Probably the cause in this case is that there are very few “food atopic” dogs, and these do not respond in 3 or 4 weeks, as in the case of enteropaths, but after months.

The data that emerged reflect the importance of communicating what results we expect to achieve and in what time frame to the owner during the clinical visit, especially if it is a specialist consultation.

It must be highlighted that the primary reasons for discontinuing the prescribed nutritional plan during the follow-up period were predominantly linked to owner compliance issues, not adverse events involving pets. Among the owners who reverted to the previous diet habit, 43% of them made this decision because of their unwillingness to proceed with the tasks that involved this nutritional plan, including timing for preparing the daily ration or the difficulties in finding the supplements. Only 24% of these 63 pet owners reported giving up on the prescribed diet due to challenges related to this food plan, while 12% were forced to abandon the homemade diet because the dogs did not like it. In the remaining cases, the reason for dropping the study was linked to the onset of new pathologies (8%) or the death of the animal (13%).

The relatively high incidence of pet owners who discharge the prescribed homemade diet may be due to a need for more profound knowledge of this nutritional approach. Clients should be guided in preparing daily rations to avoid wasting their time. Furthermore, they should also be trained to purchase the best ingredients and supplements easily, and a new and responsible culture around pet nutrition and specific needs should be created. It is worth pointing out the urgency for health-economics assessments to disclose the long-term benefits of the animals and their owners regarding wellbeing, quality of life, and related cost savings in terms of drug consumption and veterinary costs.

Among the cohort of healthy dogs that continued the homemade diet for maintenance purposes, a substantial proportion of the owners (70%) reported improvements in coat condition, with the coat becoming shinier and softer, and a decrease in evacuation frequency was reported in 47% of cases. While these positive effects are encouraging, it is essential to acknowledge that not all dogs experienced such improvements, and some developed new symptoms, particularly gastrointestinal signs, after introducing the homemade diet. This outcome points out the current challenges associated with the introduction of a new diet. However, with the nutritionist’s guidance and the right approach, these challenges can be overcome and the benefits of a homemade diet can be maximized, including its potential to prevent later food-related diseases.

Based on the opinions of dog owners, it appears that those who continued the home diet to the end were about 4 times more likely to improve palatability than those who stopped the home diet early. The dogs’ energy as observed by the owners exhibits a trend in the same direction, where those who continued the home diet to the end were about three and one-half times more likely to be more energetic. As for the coat, it shows the biggest impact, where—based on the evidence gathered—it appears that dogs who have completed the home diet are 10 times more likely to see an improvement in their coat. The variables relating to feces are not significant, indicating that—according to the interviewees—there are no major changes in either case.

Among the dogs that kept the prescribed diet and had weight loss goals, four out of six achieved their target weight and attained an appropriate BCS. These results support the potential effectiveness of homemade diets in supporting weight management objectives for overweight or obese dogs. Even in this regard, the literature is not consistent in supporting this statement. However, the authors’ clinical experience suggests that by better intercepting dogs’ food preferences and working on the volume of the cooked ingredients, it is often easier to reach the weight reduction target by enhancing the pet’s satiety level.

The most notable outcomes involved the 67 pathological cases primarily involving gastrointestinal and/or dermatological disorders. According to the owners’ responses, the prescribed homemade diet led to improvements in about 94% of the sick subjects. These results suggest that a homemade diet could be beneficial in managing certain pathological conditions, especially those associated with adverse food reactions, and this aligns with previous research that has reported favorable outcomes when dealing with homemade diets for specific medical conditions [3,13].

It is essential to underscore that the main improvements disclosed in dogs with one or more gastrointestinal issues concerned young or young adult patients (75%). For this reason, it is possible to assume that young dogs affected by various enteropathies should benefit from the homemade nutritional approach, suggesting that this type of diet should be included among the treatment strategies.

Finally, 100% of the successes gained in subjects with both gastrointestinal and dermatological disorders suggest that a tailored homemade diet might be the unique way to nutritionally treat two or more existing pathologies. With commercial pet food, veterinarians must prioritize one disorder over another. Moreover, a homemade diet overcomes allergens coming from mites or preservatives present in commercial pet foods [12,14], avoiding additional foods that may be allergenic.

Besides the new insight into owner perception of homemade diets for their dogs, some limitations should be acknowledged. One of the limitations of this prospective study is the lack of a medical evaluation at the end of the study, which may have led to underestimating the results. It should also be acknowledged that the study design did not consider a control group and a randomization, limiting the generalizability of the present findings to other dogs. 

The authors advocated for further prospective, randomized, controlled trials to confirm these conclusions and shed light on additional advantages of a personalized and well-balanced homemade diet in preventing and addressing specific medical conditions.

## 5. Conclusions

The clinical results obtained in this study are promising. Owners of dogs with diet-related diseases who chose to rely on homemade feeding and pursued this diet could witness almost complete remission of their pets’ symptoms. The personalized food plans considering the individual preferences and medical conditions of the animals, as well as the careful selection of functional ingredients and fresh foods concerning sources that might be allergenic, contributed to the success of the homemade diet regimen. Incorporating essential vitamin–mineral supplements and pathology-specific nutraceutical feeds supported the nutritional needs of dogs with underlying pathologies. The customization of the homemade diet by the nutritionist significantly improved the overall health of the dogs, both healthy and pathological, confirming the importance of a homemade cooked diet as a valuable tool in veterinary practice.

## Figures and Tables

**Table 1:** Types of diets which the animals were fed at the study beginning and types of diets in which the animals completed the study. The number of dogs fed with a mixed diet (homemade diet and commercial food) is reported in brackets.

Type of Diet	Beginning of Study	End of Study	*p* Value
Homemade diet	55 (26)	104 (24)	<0.0001
Commercial pet food diet	112	63	

**Table 2:** Reasons for nutritional consultations in the 104 dogs who completed the follow-up and in 63 dogs who did not complete the follow-up.

Referral	Number of Dogs Who Completed the Follow-Up (n = 104)	Number of Dogs Who Did Not Complete the Follow-Up (n = 63)	*p* Value
Maintenance diet	31	17	0.855
Obesity	6	3	1.000
Gastroenteropathy	36	11	0.020
Dermopathy	12	11	0.354
Gastroenteropathy + dermopathy	12	17	0.019
Various pathologies	7	4	1.000

**Table 3:** Odds ratios and significant variables in terms of palatability (*p*-value 0.003), energy (*p*-value 0.017), coat (*p*-value < 0.001), and stool frequency and consistency.

	Odds Ratio	*p*-Value	Significancy
Palatability	4.07	0.003	**
Energy	3.47	0.017	*
Coat	9.88	<0.001	***
Stool Frequency	0.01	0.93	-
Stool Consistency	0.01	0.97	-
Signif. codes: 0 ‘***’ 0.001 ‘**’ 0.01 ‘*’ 0.05 ‘ ’ 0.1 ‘ ’ 1			
Dog size, age, sex have been included in the model as control variables			

## Data Availability

Data presented in this study will be available upon request.
